# Risk of Thrombo-Embolic Events in Ovarian Cancer: Does Bevacizumab Tilt the Scale? A Systematic Review and Meta-Analysis

**DOI:** 10.3390/cancers13184603

**Published:** 2021-09-14

**Authors:** Michael Saerens, Emiel A. De Jaeghere, Heini Kanervo, Nele Vandemaele, Hannelore Denys, Eline Naert

**Affiliations:** 1 Department Medical Oncology, Ghent University Hospital, 9000 Ghent, Belgium; emiel.dejaeghere@ugent.be (E.A.D.J.); heini.kanervo@uzgent.be (H.K.); nele.vandemaele@ugent.be (N.V.); Hannelore.Denys@UGent.be (H.D.); eline.naert@uzgent.be (E.N.); 2 Laboratory of Experimental Cancer Research, Department of Human Structure and Repair, Ghent University, 9000 Ghent, Belgium; 3 Gynecological Pelvic Oncology Network (GYPON), 9000 Ghent, Belgium

**Keywords:** ovarian cancer, bevacizumab, thromboembolism, deep vein thrombosis, meta-analysis

## Abstract

**Simple Summary:**

Thromboembolic events (TEs) are the second cause of death in cancer patients. Two forms of thromboembolic events may arise: arterial, such as ischemic stroke or myocardial infarction; and venous, such as deep vein thrombosis or pulmonary embolism. Bevacizumab is a monoclonal antibody directed against vascular endothelial-derived growth factor, and is widely used in advanced ovarian cancer. However, whether bevacizumab increases the risk of thromboembolic events in ovarian cancer is matter of debate since studies have shown conflicting results. In our systematic review and meta-analysis, we included 14 trials with bevacizumab in ovarian cancer. We found that the risk of arterial thromboembolic events more than doubled with a risk ratio of 2.45. Also the risk of venous thromboembolism increased 30% with bevacizumab treatment. Bevacizumab, therefore, can be considered an additional risk factor for selecting patients for primary prophylaxis with anticoagulants.

**Abstract:**

Thromboembolic events are the second cause of death in cancer patients. In ovarian cancer, 3–10% of patients present with venous thromboembolism (VTE), but the incidence may rise to 36% along the disease course. Bevacizumab is a monoclonal antibody directed against vascular endothelial-derived growth factor, and in in vitro studies it showed a predisposition to hemostasis perturbation, including thrombosis. However, in vivo and clinical studies have shown conflicting results for its use as a treatment for ovarian cancer, so we conducted a systematic review and meta-analysis on the risk of arterial thromboembolism (ATE) and VTE in ovarian cancer patients treated with bevacizumab. The review comprised 14 trials with 6221 patients: ATE incidence was reported in 5 (4811 patients) where the absolute risk was 2.4% with bevacizumab vs. 1.1% without (RR 2.45; 95% CI 1.27–4.27, *p* = 0.008). VTE incidence was reported in 9 trials (5121 patients) where the absolute risk was 5.4% with bevacizumab vs. 3.7% without (RR 1.32; 95% CI 1.02–1.79, *p* = 0.04). Our analysis showed that the risk of arterial and venous thromboembolism increased in patients treated with bevacizumab. Thrombolic events (TEs) are probably underreported, and studies should discriminate between ATE and VTE. Bevacizumab can be considered as an additional risk factor when selecting patients for primary prophylaxis with anticoagulants.

## 1. Introduction

Thromboembolic events (TEs) frequently occur during malignancy and are the second leading cause of mortality in cancer patients [1,2,3,4]. Furthermore, TEs lead to increased morbidity, including the need for chronic anticoagulation, possible delays in delivering chemotherapy, high risk of a recurrent TE, risk of bleeding complications from anticoagulation, decreased quality of life, and consumption of health care resources [4,5]. Two categories of TEs are recognized: arterial thrombo-embolism (ATE), such as ischemic stroke or myocardial infarction; and venous thromboembolism (VTE), such as deep vein thrombosis or pulmonary embolism.

In ovarian cancer, 3–10% of patients present with a VTE at diagnosis, but the incidence increases up to 36% during treatment [6,7]. Risk factors for developing a TE may be related to the patient (e.g., age, comorbidity, immobilization), disease (e.g., compression, stage, subtype) or treatment (e.g., IV catheter, surgery), all of which contribute to the “prothrombotic” or hypercoagulable state, as defined by Virchow’s triad: stasis, hypercoagulability, and endothelial injury) [8,9].

Angiogenesis is one of the hallmarks of malignancy, and the vascular endothelial growth factor (VEGF) is one of the key promoting factors because it alters the tumor micro-environment and its cancer cells. Bevacizumab is a recombinant humanized monoclonal antibody against VEGF and is widely used in the treatment of different cancer types, either in monotherapy or in addition to chemotherapy or immunotherapy [10,11,12,13,14,15]. Bevacizumab is FDA and EMA approved for front-line treatment of ovarian cancer (OC) patients in combination with chemotherapy followed by single-agent bevacizumab for FIGO (*Fédération Internationale de Gynécologie et d’Obstétrique*) stage-III (EMA IIIB and IIIC only) and stage-IV disease based on the GOG-0218 [16] and ICON7 trial [17], both of which demonstrated a significant benefit for median progression-free survival (mPFS) for concurrence and maintenance. However, both trials failed to show an overall survival (OS) benefit [16,17,18,19]. Furthermore, bevacizumab is approved in combination with platinum-based chemotherapy in recurrent platinum-sensitive OC based on the OCEANS [20] and GOG-0213 [21] trial; and in platinum-resistant OC in combination with paclitaxel, pegylated liposomal doxorubicin (PLD), or topotecan based on the AURELIA trial [22].

Overall, bevacizumab has a manageable safety profile and is well tolerated. Typical adverse events include hypertension (17–23%; >grade 2), proteinuria (3–40% overall; 1–10% grade 3–4) and minor mucocutaneous bleeding (7–36%) [17,23,24,25]. Of special interest are delayed wound healing and gastrointestinal complications, such as perforations or fistulae. In fact, >grade 3 wound healing complications were seen with bevacizumab in 3% (GOG-0218) and 5% (ICON7), compared to 2.1 and 2.8% without bevacizumab, respectively [16,18]. For GI complications (perforations and fistulae), the incidences with bevacizumab were 1.3 and 2.8%, respectively, versus 0.4 and 1.2% without [18,23]. Therefore, treatment interruption with bevacizumab has been advised prior and after surgery, and the risk of GI complications should be carefully evaluated when bevacizumab is an option [25]. Whether bevacizumab increases the risk of thrombo-embolic events is matter of debate. 

The in vitro inhibition of the VEGF pathway led to endothelial dysfunction and facilitates vasoconstriction which are predispositions for disturbed hemostasis and vascular thrombosis [26]. Furthermore, in vivo studies on a mouse xenograft model (A549) treated with bevacizumab showed high plasminogen activator inhibitor (PAI-1) levels in plasma and increased expression in endothelial cells, platelets, leukocytes and circulating tumor cells, which resulted in increased thrombosis formation in the inferior vena cava and femoral vein [27]. Retrospective studies have suggested an increased risk of VTE with bevacizumab in the treatment of colorectal and breast cancer [28,29] as well as OC [30], but since the data from the OC studies showed conflicting results, no definite conclusions could be drawn. Therefore, we performed a systematic review and meta-analysis on the risk of thromboembolic events in OC patients treated with bevacizumab.

## 2. Materials and Methods

A systematic literature search was performed using MEDLINE, EMBASE and Cochrane Central Register of Controlled Trials (CENTRAL), with final update on 2 April 2021. The search string was built with the help of a specialized librarian using search terms related to “ovarian cancer” and bevacizumab. The detailed search syntaxes for all search engines are provided in the Supplementary Figure S1. Furthermore, we allowed the inclusion of articles using cross-references from included studies. The search was reported per Preferred Reporting Items for Systematic Reviews and Meta-Analyses (PRISMA) guidelines.

### 2.1. Study Selection

The studies that met the following criteria were eligible for inclusion in our meta-analysis: (1) prospective randomized controlled trials; (2) available in English; (3) patients with OC, including primary peritoneal cancer or cancer of the fallopian tube; and (4) randomization to treatment with or without bevacizumab, alone or in combination with chemotherapy, targeted therapy, or immunotherapy. Titles and abstracts that did not clearly meet the inclusion criteria were excluded. Full texts were obtained for the remaining records. In case a study had multiple reporting articles, the most recent and most relevant was used. Articles reporting subanalyses or exploratory analyses were excluded if they were not relevant to the assessed outcome parameter (i.e., arterial/venous thromboembolic risk). The results from all search engines were merged using RAYYAN (https://rayyan.qcri.org, accessed on 1 October 2020) for further screening and selection. Screening was done by two authors (MS and NV) based on title and abstract. Full-text analysis and study selection was done by authors MS and HK. Any disagreement was resolved by consensus. When the same patient source was included in different publications (e.g., abstract on congress and full text), the most recent and relevant was used.

### 2.2. Data Extraction 

Data were extracted by authors MS and HK, and disagreements were resolved by consensus. For all the trials we extracted the following data where possible: study characteristics (author, year of publication, journal, study phase and design); patient and tumor characteristics (study population, disease setting, tumor type/subtype, number, duration of follow-up); treatment characteristics (type of treatment, dose, duration of treatment, combination regimen); and outcome parameters (progression-free survival (PFS), OS, ATE, VTE). In case of missing or confounding data, the corresponding author was contacted.

### 2.3. Study Objectives and Statistical Analysis

The primary outcome was the incidence of venous and arterial thromboembolism in patients treated with bevacizumab versus without treatment. 

We extracted the thrombo-embolic risk for individual studies when provided and made a separate analysis for venous and arterial thrombo-embolic risk. In case of a three-arm study, we pooled the data of all bevacizumab-treated patients. Missing or confounding data were addressed to the corresponding author. Data that were not provided were left blank. 

We pooled data from individual trials and calculated the risk ratio (RR) for developing a thrombo-embolic even using RevMan 5.3, provided by the Cochrane collaboration (https://training.cochrane.org/online-learning/core-software-cochrane-reviews/revman, accessed on 9 April 2021). The summary estimates were generated using a fixed-effect model (Mantel–Haenszel method). Statistical heterogeneity was assessed with the Q-test and the I^2^ statistic. I^2^ values of 25, 50 and 75% were considered to indicate low, moderate, and high heterogeneity, respectively [31]. Risk ratios for VTE and ATE were calculated with 95% CIs for each study. We made a predefined subgroup analysis for treatment setting and dose of bevacizumab. For all the statistical analyses, a *p* < 0.05 was regarded as statistically significant, and all tests were two-sided. If >10 studies were included, a funnel plot was generated to evaluate the publication bias, and Egger’s regression method was used to test the symmetry of funnel plots. Risk of bias was assessed by two reviewers (MS and HK) using the Cochrane RoB2-tool [32]. Any disagreements were resolved by consensus.

### 2.4. Protocol Registration

Details of the protocol for this systematic review were registered with PROSPERO (www.crd.york.ac.uk/PROSPERO/display_record.asp?ID=CRD42020176635, accessed on 26 March 2020).

## 3. Results

### 3.1. Study Selection and Characteristics

**Figure 1:**
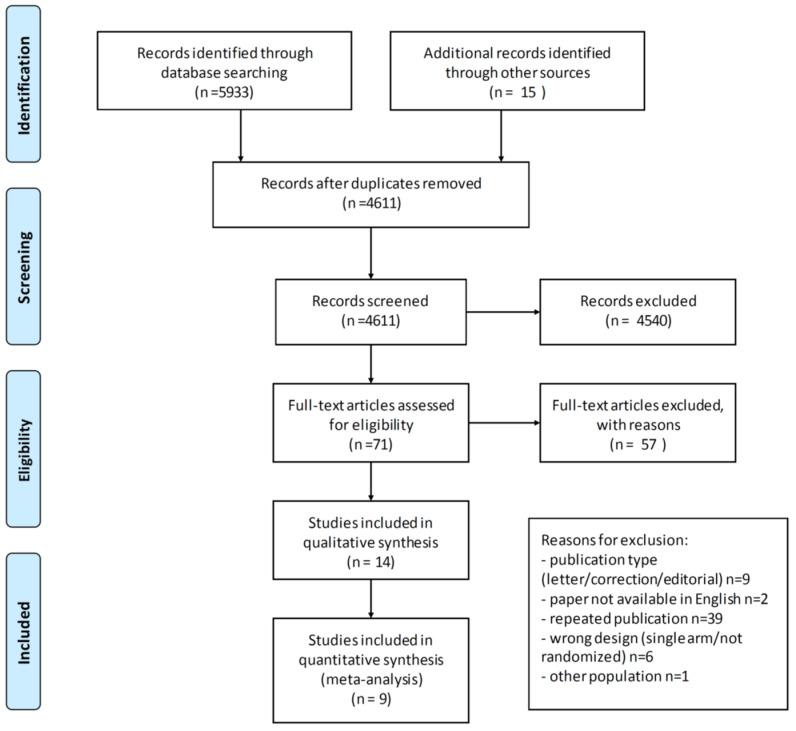
PRISMA flowchart of study selection. From: Moher D, Liberati A, Tetzlaff J, Altman DG, The PRISMA Group (2009). Preferred Reporting Items for Systematic Reviews and Meta-Analyses: The PRISMA Statement. PLoS Med 6(7): e1000097. doi:10.1371/journal.pmed1000097.

The literature search resulted in 4611 possible studies, which were screened. The selection process is illustrated in the PRISMA-flowchart in Figure 1. In all, 71 articles underwent full-text review, of which 14 were available for inclusion for a total of 6119 patients (range 50–1873). Six studies were conducted in a frontline setting [16,17,33,34,35,36], 6 trials included patients with relapsed platinum-sensitive OC [20,21,37,38,39,40] and 2 with platinum-resistant OC [22,41]. The characteristics of all trials in the qualitative analysis are provided in Table 1. ATE and VTE incidence was reported in 5 and 9 studies, respectively, and only these were included in the quantitative analysis (see Figure 1). Since <10 studies were included in the quantitative analysis, no funnel plot was generated. 

### 3.2. Arterial Thromboembolic Events

**Figure 2:**
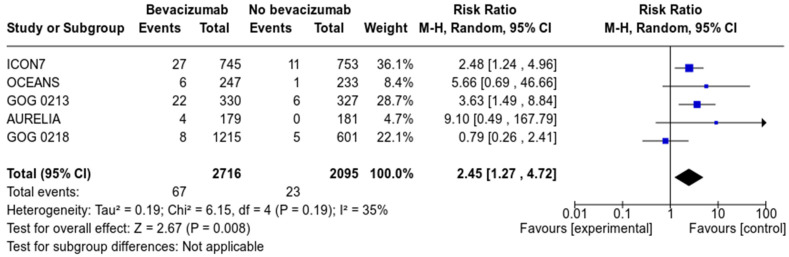
Forrest plot of arterial thrombo-embolic events (ATE).

Five studies, with a total of 4811 patients (2716 in the bevacizumab group and 2095 in the control group) reported on the incidence of arterial thromboembolism (see Figure 2). With a total number of 67 events in the bevacizumab group and 23 in the control group, we calculated that the absolute risk of arterial thromboembolic events was, respectively, 2.4 and 1.1%. Pooled analysis revealed that treatment with bevacizumab significantly increased the risk of ATE (RR 2.45; 95% CI 1.27–4.27, *p* = 0.008). Heterogeneity between studies was moderate (I^2^ = 35%). Based on subgroup analysis, this risk was present in all treatment settings: either frontline treatment, relapsed platinum-sensitive or platinum-resistant (Supplementary Figure S2). 

### 3.3. Venous Thromboembolic Events

Nine studies, with a total of 5121 patients (2882 in the Bevacizumab arm, and 2239 in the control arm), reported on the incidence of venous thromboembolism. With a total number of 155 events in the bevacizumab group and 83 in the control group, the absolute risk of development of VTE was 5.4 and 3.7%, respectively.

**Figure 3:**
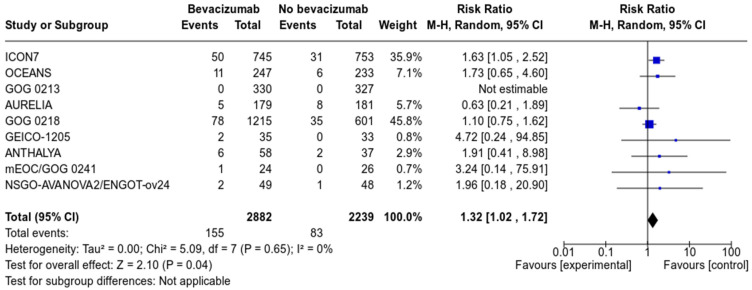
Forrest plot of venous thromboembolic events (VTE).

Pooled analysis revealed that treatment with bevacizumab increased the risk of venous thromboembolism (RR 1.32, 95% CI 1.02–1.79, *p* = 0.04) compared to no bevacizumab treatment (see Figure 3). Subgroup analysis according to treatment setting showed that the increased risk was present in the frontline setting as well as in relapsed platinum-sensitive OC (See Supplementary Figure S3). 

### 3.4. Risk of Bias

The risk of bias for 4 studies was low according to the Cochrane Risk of Bias tool [16,17,20,35]. There was some concern about 6 others because details on the randomization process and allocation concealment were lacking [22,33,34] or the outcome assessment was non-blinded [21,37,38,40,41]. Two studies [36,39] were considered high risk because of a lack of detail on randomization, outcome assessment and outcome reporting. The details are summarized in Table 2.

## 4. Discussion

To our knowledge, our study is the first meta-analysis that specifically addressed the arterial and venous thromboembolic risk of bevacizumab in ovarian cancer in various treatment settings. Based on our analysis, patients treated with bevacizumab had an increased risk of ATE and VTE, with a relative risk ratio of 2.45 (95% CI, 1.27–4.72) and 1.32 (95% CI, 1.02–1.72), respectively. However, we also found that the absolute risk of ATE and VTE was lower than expected in both groups as the incidence of ATE and VTE was, respectively, 2.4 and 5.4% in the bevacizumab group and 1.1 and 3.7% in the control group. 

Our study is the largest meta-analysis to address the thrombogenic risk of bevacizumab in ovarian cancer. We selected 14 studies with a total of 6119 patients and made a subgroup analysis according to disease setting. This was particularly interesting as the incidence of TEs may vary among tumor types and disease setting. However, we used pooled data from the trials as we did not have access to individual patient data. We could not account for possible confounding factors according to disease and patient characteristics (e.g., prolonged immobilization or poor performance status), surgical factors (e.g., surgical effort), or prophylactic use of LMWH. Furthermore, we were confronted with scarce and heterogeneous data because ATE and VTE incidence was only reported in 5 and 9 studies, respectively, of the 14 studies overall. We decided to select all prospective trials for qualitative review, including those not reporting ATEs and VTEs, to minimize the risk of selective outcome reporting and to illustrate the fact of inadequate TE reporting: five of the studies did not report any incidence of ATE or VTE (see Table 1). Of the reporting trials, five described only an advanced (grade 3 or higher) VTE [20,21,22,34,35] and four others reported all VTEs [16,17,33,38]. Moreover, in the GOG-0213 trial, there were two cases of pulmonary embolism that led to treatment discontinuation, yet these were not reported in the adverse events table as VTE [21]. In studies with bevacizumab, adequate reporting of TE complications is mandatory, distincting ATEs from VTEs. 

This retrospective series showed thromboembolic events in 12–36% of ovarian cancers [6,7,42]. In the single-arm OSCAR trial, which evaluated the use of front-line bevacizumab in advanced ovarian cancer, the incidence of TE was 9% [43]. With an absolute risk of 5.4% (with bevacizumab) and 3.7% (without bevacizumab), the incidence of VTE was lower than expected in our study. This may be related to underreporting, as illustrated above, or selection bias as patients with prior VTE or hypercoagulability might have been excluded from the cancer trials. However, these adverse events should not be neglected, as they might be life-threatening. The increased TE risk, as documented in our analysis, calls for clinical vigilance for thromboembolic complications and the collection of real-world data, to further determine TE incidence and risk factors.

Previous meta-analyses showed conflicting results for the risk of TE with bevacizumab (see Table 3). In 2007, a meta-analysis including 1745 patients with advanced breast, lung and colorectal cancer showed an increased risk of ATE yet no increased risk of VTE [44]. These results were contested by Nalluri et al., who performed a larger meta-analysis in 7956 patients with advanced breast, lung, colorectal, renal or pancreatic cancer and found an increased risk of low-grade and high-grade VTE independent of the bevacizumab dose [45]. However, this study was criticized as it included studies that did not distinguish between venous and arterial events [46,47,48]. Two other meta-analyses with mixed cancer types confirmed the increased risk of ATE with bevacizumab with a RR of 1.44 (95% CI 1.08–1.91) and 1.46 (95% CI, 1.11–1.93), respectively, but these studies did not investigate the risk for VTE [49,50]. Another meta-analysis in breast cancer found no increased risk for VTE (RR 1.02, 95% CI 0.70–1.61) or ATE (RR 1.49; 95% CI, 0.70–3.19) [51]. The largest meta-analysis, which included 20,500 patients with multiple cancer types found an increased risk for ATE and VTE, but no subgroup analysis for cancer type was made [52]. In advanced lung cancer, a Chinese meta-analysis found an increased risk for all TEs (RR 1.74; 95% CI, 1.15–2.62) but did not make a subanalysis for VTE or ATE [53]. The increased risk was driven by the high-dose group (15 mg/m^2^ Q3w), whereas the result in the low-dose group (7.5 mg/m^2^) was not significant [53].

In ovarian cancer, six meta-analyses investigated the benefit and harm of angiogenesis inhibitors, including thromboembolic events (see Table 4). Five found an increased risk of ATE with bevacizumab with a RR ranging from 2.27 to 4.84 [55,56,57,58,59]. However, other angiogenesis inhibitors besides bevacizumab were evaluated. Another meta-analysis found an increased risk of all TEs but did not distinguish between ATE and VTE [60]. Our study is in line with the largest meta-analysis regarding cardiovascular adverse events with bevacizumab by Totzeck et al. [52], and the study by Wu et al. [59], further establishing the increased risk of VTE and ATE in ovarian cancer patients treated with bevacizumab. Based on these data, bevacizumab treatment can be considered a risk factor for VTE and ATE development. 

### Implications for Clinical Practice

The burden of thromboembolic complications in ovarian cancer is high, and there is a clinical need to investigate relevant risk factors for TEs to define possible prevention strategies for TE development in OC. 

The Khorana risk score (KRS) was the first risk assessment model to identify ambulatory patients at risk for VTE, with a score of >3 as high risk [61]. Several other models have been designed to improve the VTE risk discrimination capacity [62,63,64,65], yet they have to be validated before routine introduction [66]. To date, the KRS remains the most validated for risk assessment and guidance for TE prophylaxis, either with low-molecular-weight heparin (LMWH) [67,68,69] or direct oral anticoagulants (DOACs) [70,71]. There are no direct comparisons between LMWH and a DOAC as a preventive strategy; however, DOACs are considered more convenient because they do not require daily injections of LMWH. In a recent meta-analysis, DOACs had lower 6-month recurrent VTE compared to LMWH (RR 0.65, 95% CI 0.42–1.01), at the price of increased major bleeding (RR 1.74; 95% CI 1.05–2.88) and non-major bleeding (RR 2.31; 95% CI 0.85–6.28) for patients receiving DOACs. There was no difference in mortality (RR 1.03; 95%CI 0.85–1.26) [72]. 

Current guidelines recommend primary prophylaxis for hospitalized patients and prolonged prophylaxis after surgery. In ambulatory settings, prophylaxis is recommended for high-risk patients (Khorana risk score > 2) [73,74,75,76] although studies with LMWH used a KRS of >3 as this resulted in a larger reduction in VTE incidence [77] and yielded greater cost-effectiveness [78]. Primary prophylaxis could be considered for newly diagnosed advanced OC patients with BMI > 35 kg/m^2^, hemoglobin < 10 g/dL, leukocytosis > 11 × 10^6^/µL or thrombocytosis > 350,000/µL. When weighing risks and benefits, treatment with bevacizumab can be considered as an additional risk factor besides others such as ascites, clear cell histology or a prior history of VTE [79].

## 5. Conclusions

In ovarian cancer patients, an increased risk of ATE and VTE was observed in treatment with bevacizumab. The incidence of TEs was probably underreported, and arterial and venous TE should be described separately. Caution should be made when initiating bevacizumab in patients at risk for TE. Primary prophylaxis of VTE with LMWH or DOACs, based on the Khorana risk score, may reduce the TE burden in OC. When selecting patients for primary prophylaxis, treatment with bevacizumab should be considered as an additional risk factor for VTE development.

## Figures and Tables

**Table 1:** Overview of the studies with and without bevacizumab in ovarian cancer.

Study Name Author, Year [Ref]	Study Population, Number	Regimen	mPFS (Months) *p* Value	mOS (Months) *p* Value	VTE n/N (%)	ATE n/N (%)
GOG-0218 Tewari, 2018 [16]	Frontline stage III (incomplete resection) and stage IV (all) N = 1873	A: C + P + pbo x6 B: C + P + bev x6 C: C + P + bev x6 → bev x16	A: 10.3 m B: 11.3 m C: 14.1 m	A: 41.1 m B: 40.8 m C: 43.3 m	A: 35/601 (5.8%) B: 36/607 (5.9%) C: 42/608 (6.9%)	A: 5/601 (0.7%) B: 4/607 (0.7%) C: 4/608 (0.7%)
ICON-7 Perren, 2011 [17]	Frontline stage I-IIa (high risk) OR stage IIb-IV N = 1498	A: C + P + pbo x6 → pbo x10 B: C + P + bev^1^ x6 → bev^1^ x10	A: 17.5 m B: 19.9 m	A: 58.6 m B: 58.0 m	A: 31/753 (4.1%) B: 50/745 (6.7%)	A: 11/753 (1.4%) B: 27/745 (3.6%)
ANTHALYA Joly, 2017 [33]	Frontline stage IIIc-IV neoadjuvant N = 99	A: C + P x4 → IDS → C + P + bev x1 → bev x16 B:(C + P) x4 + bev x3 →IDS →(C + P) x2 + bev x1 → bev x16	A: 21.2 m [14.5–26.7] B: 23.5 m [18.5–30.6]	NA	A: 2/37 (5%) B: 6/58 (11%)	NA
GEICO-1205 Garcia-Garcia, 2019 [34]	Frontline FIGO IIIc-IV OC neoadjuvant N = 68	A: C + P x4 → IDS →C + P + bev x3 → bev x15 B: C + P + bev x4 → IDS → C + P + bev x3 → bev x15	A: 20.1 m B: 20.4 m	NA	A: 0/33 (0%) * B: 2/35 (5.7%) *	NA
mEOC/GOG0421 Gore, 2019 [35]	Frontline mucinous stage II-IV or relapsed stage I N = 50	A: C + P x6 vs. B: Ox + Cap x6 vs. C: C + P + Bev x6 → bev x12 vs. D: Ox + Cap + Bev x6 → bev x12	A&B: 8.1 m C&D: 18.1 m	A&B: 32.7 m C&D: 27.7 m	A&B: 0/26 (0%) * C&D:1/24 (4.1%) *	NA
Zhang, 2020 [36]	Frontline stage I-III N = 100	A: C B: Npl85 + bev^1^	A: NA B: NA	A: NA B: NA	A: NA B: NA	A: NA B: NA
OCEANS Aghajanian, 2015 [20]	Relapsed platinum-sensitive N = 484	A: C + G + pbo x6 B: C + G + bev x6 → bev maintenance	A: 8.4 m B: 12.4 m	A: 32.9 m B: 33.6 m	A: 6/233 (2.6%) * B:11/247 (4.5%) *	A: 1/233 (0.4%) B: 6/247 (2.4%)
GOG-0213 Coleman, 2017 [21]	Relapsed platinum-sensitive N = 674	A: C + P B: C + P + bev x6 → bev maintenance ***	A: 10.4 m B: 13.8 m	A: 37.3 m B: 42.2 m	A: 0/327 (0%) B: 0/330 (0%)	A: 6/327 (1.8%) B: 22/330 (6.6%)
MITO16B-MaNGO OV2B-ENGOT OV17 Pignata, 2021 [37]	Relapsed platinum sensitive, prior bevacizumab N = 406	A: C + P or C + G or C + PLD B: C + P or C + G or C + PLD + bev^2^	A: 8.8 m B: 11.8 m	A: 27.1 m B: 26.7 m	NA **	NA **
NSGO-AVANOVA2/ ENGOT-OV24 Mirza, 2019 [38]	Relapsed platinum sensitive N = 97	A: niraparib B: niraparib + bev	A: 5.5 m B: 11.9 m	NA	A: 1/48 (2.2%) B: 2/49 (4.2%)	NA
Cong, 2019 [39]	Relapsed platinum sensitive N = 164	A: C + P B: C + P + bev^2^	A: 6.7 m B: 9.3 m	A: 12.5 m B: 18.5 m	NA	NA
AURELIA Pujade-Lauraine, 2014 [22]	Relapsed platinum resistant N = 360	A: wP or Tp or PLD B: wP or Tp or PLD + bev	A: 3.4 m B: 6.7 m	A: 13.3 m B: 16.6 m	A: 8/181 (4.4%) * B: 5/179 (2.8%) *	A: 0/181 (0%) B: 4/179 (2.2%)
Liu, 2019 [41]	Relapsed platinum resistant N = 86	A: ABP B: ABP + bev^1^	A: 6.7 m B: 8.9 m	A: 12.7 m B: 16.3 m	NA	NA
Zhang, 2019 [40]	Relapsed platinum sensitive N = 160	A: DTx + Npl80 B: DTx + Npl80 + bev^1^	A: 8.6 m B: 12.2 m	A: 17.7 m B: 22.5 m	NA	NA

ABP: albumin-bound paclitaxel 135–175 mg/m^2^ Q3w x6; ATE: arterial thromboembolic events; bev = bevacizumab 15 mg/kg IV Q3w; bev^1^ = bevacizumab 7.5 mg/kg IV Q3w; bev^2^ = bevacizumab 10 mg/kg IV Q2w or 15 mg/kg Q3w; C: carboplatin 400 mg/m^2^ Q3w x3; C + G: Carboplatin AUC4 Q3w + gemcitabin 1250 mg/m^2^ d1 + 8; C + P: carboplatin AUC5-6 Q3w + paclitaxel 175 mg/m^2^ Q3w x6; C + PLD: carboplatin AUC5 + pegylated liposomal doxorubicin 30 mg/m^2^ Q4w; DTX: Docetaxel 75 mg/m^2^; PLD: pegylated liposomal doxorubicin 40 mg/m^2^ Q4w; IDS: interval debulking surgery; mPFS: median progression-free survival expressed in months; mOS: median overall survival expressed in months; NA: not available; Npl80: nedaplatin 80 mg/m^2^ Q3w; Npl85: nedaplatin 85 mg/m^2^ Q3w x6; niraparib: 300 mg once daily; Ox-Cap: oxaliplatin 130 mg/m^2^ intravenous d1 Q2w + capecitabine 850 mg/m^2^ orally bid d1–14; pbo: placebo; Tp: Topotecan 4 mg/m^2^ d1–8–15 Q3w; VTE: venous thromboembolism (all grades unless otherwise specified); wP: Paclitaxel 80 mg/m^2^ d1–8–15–22 Q4w. * Grade ≥ 3 VTE. ** all thromboembolic events: 5/200 (A) vs. 6/200 (B) *** 2 × 2 design: surgery vs. no surgery; bevacizumab vs. no bevacizumab. Data shown with/without bevacizumab. Surgery analysis was done in a separate publication.

**Table 2:** Risk of Bias analysis of the included studies, according to the Cochrane Risk of Bias (RoB)-2 tool [32].

Study Name Author, Year [Ref]	Domain 1 Randomization Bias	Domain 2 Intervention Bias	Domain 3 Bias in Missing Outcome Data	Domain 4 Bias in Measurement of Outcome	Domain 5 Bias in Selection of the Reported Result	Overall Bias
GOG-0218 Tewari, 2018 [16]	Low risk	Low risk	Low risk	low risk	Low risk	Low risk
ICON-7 Perren, 2011 [17]	Low risk	Low risk	Low risk	low risk	Low risk	Low risk
ANTHALYA Joly, 2017 [33]	Some concerns	Some concerns	Low risk	Some concerns	Low risk	Some concerns
GEICO-1205 Garcia-Garcia, 2019 [34]	Some concerns	Some concerns	Low risk	Some concerns	Low risk	Some concerns
mEOC/GOG0421 Gore, 2019 [35]	Some concerns	Low risk	Low risk	low risk	Low risk	Low risk
Zhang, 2020 [36]	Some concerns	High risk	Low risk	High risk	Low risk	High risk
OCEANS Aghajanian, 2015 [20]	Low risk	Low risk	Low risk	low risk	Low risk	Low risk
GOG-0213 Coleman, 2017 [21]	Low risk	Low risk	Some concerns	Some concerns	Some concerns	Some concerns
MITO16B-MaNGO OV2B-ENGOT OV17 Pignata, 2021 [37]	Some concerns	Low risk	Low risk	Some concerns	Low risk	Some concerns
NSGO-AVANOVA2/ ENGOT-OV24 Mirza, 2019 [38]	Some concerns	Low risk	Low risk	Some concerns	Low risk	Some concerns
Cong, 2019 [39]	Some concerns	High risk	Low risk	High risk	Low risk	High risk
AURELIA Pujade-Lauraine, 2014 [22]	Some concerns	Low risk	Low risk	Some concerns	Low risk	Some concerns
Liu, 2019 [41]	Low risk	Some concerns	Low risk	Some concerns	Low risk	Some concerns
Zhang, 2019 [40]	Low risk	Some concerns	Low risk	Some concerns	Low risk	Some concerns

**Table 3:** Previous meta-analyses on the thromboembolic risk with bevacizumab in various tumor types.

Author, Year [Ref]	Tumor Types	Total Included Patients	All TE RR (95% CI)	VTE RR (95% CI)	ATE RR (95% CI)
Scappaticci, 2007 [44]	Lung, colorectal, breast	1745	NA	0.89 (0.66–1.20) *p* = 0.44	1.8 (0.94–3.33) *p* = 0.076
Nalluri, 2008 [45]	Lung, colorectal, breast, renal, pancreatic	7956	NA	1.33 (1.13–1.56) *p* < 0.001	NA
Ranpura, 2010 [49]	Lung, colorectal, breast, renal, pancreatic	12,617	NA	NA	1.44 (1.08–1.91) *p* = 0.013
Azzi, 2010 [50]	not specified	13,026	NA	NA	1.46 (1.11–1.93) *p* = 0.007
Hurwitz, 2011 [54]	Lung, colorectal, breast, renal, pancreatic	6055	NA	0.91 (0.77–1.06) *p* = 0.23	NA
Cortes, 2012 [51]	breast	3784	NA	1.02 (0.70–1.61) *p* = 0.78	1.49 (0.70–3.19) *p* = 0.30
Totzeck, 2017 [52]	Lung, colorectal, breast, renal, ovarian, gastric	20,500	NA	1.29 (1.13–1.48) *p* = 0.0001	1.37 (1.10–1.70) *p* = 0.004
Li, 2018 [53]	lung	3555	1.74 (1.15–2.62) *p* = 0.008	NA	NA

VTE: venous thromboembolism; ATE: arterial thromboembolism; RR: risk ratio; CI: confidence interval; NA: not available.

**Table 4:** Meta-analyses on the thromboembolic risk with various angiogenesis inhibitors in ovarian cancer.

Author, Year [Ref]	Therapeutic Agents Included	Total Included Patients	All TE RR (95% CI)	VTE RR (95% CI)	ATE RR (95% CI)
Zhou, 2013 [55]	bevacizumab	3621	NA	1.32 (0.99–1.75) *p* = 0.054	2.29 (1.33–3.75) *p* < 0.03
Wang, 2014 [60]	bevacizumab	3608	1.85 (1.18–2.91)	NA	NA
Li, 2015 [56]	bevacizumab	3621	NA	NA	2.33 (1.34–4.03) *p* = 0.003
Yi, 2017 [57]	bevacizumab	3211	NA	NA	4.84 (1.24–12.98) *p* = 0.03
Wu, 2017 [59]	bevacizumab	4994	NA	1.43 (1.04–1.96) *p* = 0.03	2.39 (1.39–4.10) *p* = 0.002
Wang, 2018 [58]	Bevacizumab, sorafenib, nintedanib, pazopanib, aflibercept	8721	NA	1.08 (0.79–1.48)	2.27 (1.34–3.84)
Our analysis	bevacizumab	6119	NA	1.32 (1.02–1.78) *p* = 0.04	2.45 (1.27–4.72) *p* = 0.009

VTE: venous thromboembolism; ATE: arterial thromboembolism; RR: risk ratio; CI: confidence interval; NA: not available.

## Supplementary Materials

The following are available online at https://www.mdpi.com/article/10.3390/cancers13184603/s1, Figure S1: detailed search terms and syntax, Figure S2: Forrest plots of arterial thromboembolism per disease setting, Figure S3: Forrest plots of venous thromboembolism risk per disease setting.
