# Supplementary material for: Risk of Thrombo-Embolic Events in Ovarian Cancer: Does Bevacizumab Tilt the Scale? A Systematic Review and Meta-Analysis
Source: Cancers (Basel). 2021 Sep 14;13(18):4603. doi: 10.3390/cancers13184603 (PMC8464807; doi:10.3390/cancers13184603)

Supplementary appendix:

S1: detailed overview of used search terms and search strategy

S2: Forrest plots of the risk of arterial thromboembolic events (ATE) per disease setting

S3: Forrest plots of the risk of venous thromboembolic events (VTE) per disease setting

S1: detailed overview of used search terms and search strategy

pubmed	
Concept 1: OVARIAN CANCER	“Ovarian Neoplasms”[Mesh] OR “cancer of the ovar*”[TIAB] OR “cancers of the ovar*”[TIAB] OR “carcinoma of the ovar*”[TIAB] OR “carcinomas of the ovar*”[TIAB] OR “Epithelial Ovarian Carcinoma*”[TIAB] OR “epithelial ovarian cancer*”[TIAB] OR “ovarian cancer*”[TIAB] OR “ovarian carcinoma”[TIAB] OR “ovarian neoplas*”[TIAB] OR “ovarian tumor*”[TIAB] OR “ovarian tumour*”[TIAB] OR “ovary cancer*”[TIAB] OR “ovary carcinoma”[TIAB] OR “ovary neoplasm*”[TIAB] OR “ovary tumor*”[TIAB] OR “ovary tumour*”[TIAB] OR “tumor of the ovar*”[TIAB] OR “tumour of the ovar*”[TIAB] OR “Fallopian Tube Neoplasms”[Mesh] OR “cancer of the fallopian tube”[TIAB] OR “carcinoma of the fallopian tube”[TIAB] OR “fallopian tube cancer*”[TIAB] OR “fallopian tube carcinoma*”[TIAB] OR “fallopian tube neoplas*”[TIAB] OR “fallopian tube tumor*”[TIAB] OR “fallopian tube tumour*”[TIAB] OR “tumor of the fallopian tube”[TIAB] OR “tumour of the fallopian tube”[TIAB] OR “tumors of the fallopian tube”[TIAB] OR “tumours of the fallopian tube”[TIAB] OR “primary peritoneal neoplas*”[TIAB] OR “primary peritoneal cancer*”[TIAB] OR “primary peritoneal carcinoma*”[TIAB] OR “primary peritoneal neoplas*”[TIAB] OR “primary peritoneal tumor*”[TIAB] OR “primary peritoneal tumour*”[TIAB] OR “primary cancer of the peritoneum”[TIAB] OR “primary cancers of the peritoneum”[TIAB] OR “primary carcinoma of the peritoneum”[TIAB] OR “primary carcinomas of the peritoneum”[TIAB] OR “primary tumor of the peritoneum”[TIAB] OR “primary tumors of the peritoneum”[TIAB] OR “primary tumour of the peritoneum”[TIAB] OR “primary tumours of the peritoneum”[TIAB] OR “primary carcinoma of the peritoneum”[TIAB] OR “primary carcinomas of the peritoneum”[TIAB] OR “primary peritoneum cancer*”[TIAB] OR “primary peritoneum carcinoma*”[TIAB] OR “primary peritoneum tumor*”[TIAB] OR “primary peritoneum tumour*”[TIAB] OR “cancer of the uterine tube”[TIAB] OR “carcinoma of the uterine tube”[TIAB] OR “uterine tube cancer*”[TIAB] OR “uterine tube carcinoma*”[TIAB] OR “uterine tube neoplas*”[TIAB] OR “uterine tube tumor*”[TIAB] OR “uterine tube tumour*”[TIAB] OR “tumor of the uterine tube”[TIAB] OR “tumour of the uterine tube”[TIAB] OR “tumors of the uterine tube”[TIAB] OR “tumours of the uterine tube”[TIAB]
Concept 2: BEVACIZUMAB	“bevacizumab”[Mesh] OR bevacizumab[TIAB] OR “rhuMAb VEGF”[TIAB] OR avastin[TIAB] OR “abp 215”[TIAB] OR abp215[TIAB] OR ainex[TIAB] OR altuzan[TIAB] OR "ask b1202"[TIAB] OR askb1202[TIAB] OR “bat 1706”[TIAB] OR bat1706[TIAB] OR “bcd 021”[TIAB] OR bcd021[TIAB] OR “bevacizumab awwb”[TIAB] OR “bevacizumab beta”[TIAB] OR “bevacizumab bvzr”[TIAB] OR “bevacizumab-awwb”[TIAB] OR “bevacizumab-bvzr”[TIAB] OR bevax[TIAB] OR “bevz 92”[TIAB] OR “bevz92”[TIAB] OR “bi 695502”[TIAB] OR “bi695502”[TIAB] OR bryxta[TIAB] OR “chs

	5217"[TIAB] OR chs5217[TIAB] OR "ct p16"[TIAB] OR ctp16[TIAB] OR "fkb 238"[TIAB] OR fkb238[TIAB] OR "hd 204"[TIAB] OR hd204[TIAB] OR "hlx 04"[TIAB] OR hlx04[TIAB] OR krabeva[TIAB] OR kyomarc[TIAB] OR "mb 02"[TIAB] OR mb02[TIAB] OR "mil 60"[TIAB] OR mil60[TIAB] OR mvasi[TIAB] OR "myl 14020"[TIAB] OR "myl 1402o"[TIAB] OR myl14020[TIAB] OR "myl1402o"[TIAB] OR "nsc 704865"[TIAB] OR "nsc704865"[TIAB] OR "ons 1045"[TIAB] OR "ons 5010"[TIAB] OR "ons1045"[TIAB] OR "ons5010"[TIAB] OR "pf 06439535"[TIAB] OR "pf 6439535"[TIAB] OR "pf06439535"[TIAB] OR "pf6439535"[TIAB] OR "ql 1101"[TIAB] OR "ql1101"[TIAB] OR "rg 435"[TIAB]OR "rg435"[TIAB] OR "ro 4876646"[TIAB] OR ro4876646[TIAB] OR "sb 8"[TIAB] OR "sb8"[TIAB] OR "stc 103"[TIAB] OR "stc103"[TIAB] OR zirabev[TIAB]
Combination of concepts	"#1 AND #2"

EMBASE	
Concept 1: OVARIAN CANCER	'ovary cancer'/exp OR ovary tumor/exp OR 'cancer of the ovar*':ab,ti OR 'cancers of the ovar*':ab,ti OR 'carcinoma of the ovar*':ab,ti OR 'carcinomas of the ovar*':ab,ti OR 'epithelial ovarian carcinoma*':ab,ti OR 'epithelial ovarian cancer*':ab,ti OR 'ovarian cancer*':ab,ti OR 'ovarian carcinoma':ab,ti OR 'ovarian neoplasm*':ab,ti OR 'ovarian tumor*':ab,ti OR 'ovarian tumour*':ab,ti OR 'ovary cancer*':ab,ti OR 'ovary carcinoma*':ab,ti OR 'ovary neoplas*':ab,ti OR 'ovary tumor*':ab,ti OR 'ovary tumour*':ab,ti OR 'tumour of the ovar*:ab,ti' OR 'tumor of the ovar*':ab,ti OR 'cancer of the fallopian tube':ab,ti OR 'carcinoma of the fallopian tube':ab,ti OR 'fallopian tube cancer*':ab,ti OR 'fallopian tube carcinoma*':ab,ti OR 'fallopian tube neoplas*:ab,ti' OR 'fallopian tube tumor*':ab,ti OR 'fallopian tube tumour':ab,ti OR 'tumor of the fallopian tube':ab,ti OR 'tumour of the fallopian tube':ab,ti OR 'tumors of the fallopian tube':ab,ti OR 'tumours of the fallopian tube':ab,ti OR 'primary peritoneal carcinoma'/exp OR 'primary peritoneal cancer*':ab,ti OR 'primary peritoneal carcinoma*':ab,ti OR 'primary perotineal neoplas*':ab,ti OR 'primary peritoneal tumor*':ab,ti OR 'primary peritoneal tumour*':ab,ti OR 'primary cancer of the peritoneum':ab,ti OR 'primary cancers of the peritoneum':ab,ti OR 'primary carcinoma of the peritoneum':ab,ti OR 'primary carcinomas of the peritoneum':ab,ti OR 'primary tumor of the peritoneum':ab,ti OR 'primary tumors of the peritoneum':ab,ti OR 'primary tumour of the peritoneum':ab,ti OR 'primary

	tumour of the peritoneum':ab,ti OR 'primary peritoneum cancer*':ab,ti OR 'primary peritoneum carcinoma*':ab,ti OR 'primary peritoneum tumor*':ab,ti OR 'primary peritoneum tumour*':ab,ti OR 'cancer of the uterine tube':ab,ti OR 'carcinoma of the uterine tube':ab,ti OR 'uterine tube cancer*':ab,ti OR 'uterine tube carcinoma*':ab,ti OR 'uterine tube neoplas*:ab,ti' OR 'uterine tube tumor*':ab,ti OR 'uterine tube tumour':ab,ti OR 'tumor of the uterine tube':ab,ti OR 'tumour of the uterine tube':ab,ti OR 'tumors of the uterine tube':ab,ti OR 'tumours of the uterine tube':ab,ti
Concept 2: BEVACIZUMAB	bevacizumab/exp OR 'bevacizumab':ab,ti OR avastin:ab,ti OR 'rhuMAb VEGF':ab,ti OR 'abp 215':ab,ti OR abp215:ab,ti OR ainex:ab,ti OR altuzan:ab,ti OR 'ask b1202':ab,ti OR askb1202:ab,ti OR 'bat 1706':ab,ti OR bat1706:ab,ti OR 'bcd 021':ab,ti OR bcd021:ab,ti OR 'bevacizumab awwb':ab,ti OR 'bevacizumab beta':ab,ti OR 'bevacizumab bvzr':ab,ti OR 'bevacizumab-awwb':ab,ti OR 'bevacizumab-bvzr':ab,ti OR bevax:ab,ti OR 'bevz 92':ab,ti OR 'bevz92':ab,ti OR 'bi 695502':ab,ti OR 'bi695502':ab,ti OR bryxta:ab,ti OR 'chs 5217':ab,ti OR chs5217:ab,ti OR 'ct p16':ab,ti OR ctp16:ab,ti OR 'fkb 238':ab,ti OR fkb238:ab,ti OR 'hd 204':ab,ti OR hd204:ab,ti OR 'hlx 04':ab,ti OR hlx04:ab,ti OR krabeva:ab,ti OR kyomarc:ab,ti OR 'mb 02':ab,ti OR mb02:ab,ti OR 'mil 60':ab,ti OR mil60:ab,ti OR mvasi:ab,ti OR 'myl 14020':ab,ti OR 'myl 1402o':ab,ti OR myl14020:ab,ti OR 'myl1402o':ab,ti OR 'nsc 704865':ab,ti OR 'nsc704865':ab,ti OR 'ons 1045':ab,ti OR 'ons 5010':ab,ti OR 'ons1045':ab,ti OR 'ons5010':ab,ti OR 'pf 06439535':ab,ti OR 'pf 6439535':ab,ti OR 'pf06439535':ab,ti OR 'pf6439535':ab,ti OR 'ql 1101':ab,ti OR 'ql1101':ab,ti OR 'rg 435':ab,ti OR 'rg435':ab,ti OR 'ro 4876646':ab,ti OR ro4876646:ab,ti OR 'sb 8':ab,ti OR 'sb8':ab,ti OR 'stc 103':ab,ti OR 'stc103':ab,ti OR zirabev:ab,ti

CENTRAL	
1 OVARIAN CANCER MeSH	MeSH descriptor: [Carcinoma, ovarian epithelial] explode all trees
2 OVARIAN CANCER	(Ovarian Neoplasms):MeSH OR (cancer of the ovar*:ti,ab,kw) OR (cancers of the ovar:ti,ab,kw) OR (carcinoma of the ovar*:ti,ab,kw) OR (carcinomas of the ovar*:ti,ab,kw) OR (Epithelial Ovarian Carcinoma*:ti,ab,kw) OR (epithelial ovarian cancer*:ti,ab,kw) OR (ovarian cancer*:ti,ab,kw) OR (ovarian carcinoma:ti,ab,kw) OR (ovarian neoplas*:ti,ab,kw) OR (ovarian tumor*:ti,ab,kw) OR (ovarian tumour*:ti,ab,kw) OR (ovary cancer*:ti,ab,kw) OR (ovary carcinoma:ti,ab,kw) OR (ovary neoplasm*:ti,ab,kw) OR (ovary tumor*:ti,ab,kw) OR (ovary tumour*:ti,ab,kw) OR (tumor of the ovar*:ti,ab,kw) OR (tumour of the ovar*:ti,ab,kw) OR (cancer of the fallopian tube:ti,ab,kw) OR (carcinoma of the fallopian tube:ti,ab,kw) OR (fallopian

	tube cancer*:ti,ab,kw) OR (fallopian tube carcinoma*:ti,ab,kw) OR (fallopian tube neoplas*:ti,ab,kw) OR (fallopian tube tumor*:ti,ab,kw) OR (fallopian tube tumour*:ti,ab,kw) OR (tumor of the fallopian tube:ti,ab,kw) OR (tumour of the fallopian tube:ti,ab,kw) OR (tumors of the fallopian tube:ti,ab,kw) OR (tumours of the fallopian tube:ti,ab,kw) OR (primary peritoneal neoplas*:ti,ab,kw) OR (primary peritoneal cancer*:ti,ab,kw) OR (primary peritoneal carcinoma*:ti,ab,kw) OR (primary peritoneal neoplas*:ti,ab,kw) OR (primary peritoneal tumor*:ti,ab,kw) OR (primary peritoneal tumour*:ti,ab,kw) OR (primary cancer of the peritoneum:ti,ab,kw) OR (primary cancers of the peritoneum:ti,ab,kw) OR (primary carcinoma of the peritoneum:ti,ab,kw) OR (primary carcinomas of the peritoneum:ti,ab,kw) OR (primary tumor of the peritoneum:ti,ab,kw) OR (primary tumors of the peritoneum:ti,ab,kw) OR (primary tumour of the peritoneum:ti,ab,kw) OR (primary tumours of the peritoneum:ti,ab,kw) OR (primary carcinoma of the peritoneum:ti,ab,kw) OR (primary carcinomas of the peritoneum:ti,ab,kw) OR (primary peritoneum cancer*:ti,ab,kw) OR (primary peritoneum carcinoma*:ti,ab,kw) OR (primary peritoneum tumor*:ti,ab,kw) OR (primary peritoneum tumour*:ti,ab,kw)
3 BEVACIZUMAB	bevacizumab:ab,ti,kw OR avastin:ab,ti,kw OR (rhuMAb VEGF):ab,ti,kw OR (abp 215):ab,ti,kw OR abp215:ab,ti,kw OR ainex:ab,ti,kw OR altuzan:ab,ti,kw OR (ask b1202):ab,ti,kw OR askb1202:ab,ti,kw OR (bat 1706):ab,ti,kw OR bat1706:ab,ti,kw OR (bcd 021):ab,ti,kw OR bcd021:ab,ti,kw OR (bevacizumab awwb):ab,ti,kw OR (bevacizumab beta):ab,ti,kw OR (bevacizumab bvzr):ab,ti,kw OR (bevacizumab-awwb):ab,ti,kw OR (bevacizumab-bvzr):ab,ti,kw OR bevax:ab,ti,kw OR (bevz 92):ab,ti,kw OR (bevz92):ab,ti,kw OR (bi 695502):ab,ti,kw OR (bi695502):ab,ti,kw OR bryxta:ab,ti,kw OR (chs 5217):ab,ti,kw OR chs5217:ab,ti,kw OR (ct p16):ab,ti,kw OR ctp16:ab,ti,kw OR (fkb 238):ab,ti,kw OR fkb238:ab,ti,kw OR (hd 204):ab,ti,kw OR hd204:ab,ti,kw OR (hlx 04):ab,ti,kw OR hlx04:ab,ti,kw OR krabeva:ab,ti,kw OR kyomarc:ab,ti,kw OR (mb 02):ab,ti,kw OR mb02:ab,ti,kw OR (mil 60):ab,ti,kw OR mil60:ab,ti,kw OR mvasi:ab,ti,kw OR (myl 14020):ab,ti,kw OR (myl 1402o):ab,ti,kw OR myl14020:ab,ti,kw OR (myl1402o):ab,ti,kw OR (nsc 704865):ab,ti,kw OR (nsc704865):ab,ti,kw OR (ons 1045):ab,ti,kw OR (ons 5010):ab,ti,kw OR (ons1045):ab,ti,kw OR (ons5010):ab,ti,kw OR (pf 06439535):ab,ti,kw OR (pf 6439535):ab,ti,kw OR (pf06439535):ab,ti,kw OR (pf6439535):ab,ti,kw OR (ql 1101):ab,ti,kw OR (ql1101):ab,ti,kw OR (rg 435):ab,ti,kw OR (rg435):ab,ti,kw OR (ro 4876646):ab,ti,kw OR ro4876646:ab,ti,kw OR (sb 8):ab,ti,kw OR (sb8):ab,ti,kw OR (stc 103):ab,ti,kw OR (stc103):ab,ti,kw OR zirabev:ab,ti
4 BEVACIZUMAB	MeSH descriptor: [Bevacizumab] explode all trees
Combination	(#1 OR #2) AND (#3 OR #4)

Figure S2: Forrest plots of the risk of arterial thromboembolic events (ATE) per disease setting

S2A: ATE risk in all populations

Study or Subgroup	Bevacizumab Events	Bevacizumab Total	No bevacizumab Events	No bevacizumab Total	Weight	Risk Ratio M-H, Random, 95% CI
ICON7	27	745	11	753	36.1%	2.48 [1.24 , 4.96]
OCEANS	6	247	1	233	8.4%	5.66 [0.69 , 46.66]
GOG 0213	22	330	6	327	28.7%	3.63 [1.49 , 8.84]
AURELIA	4	179	0	181	4.7%	9.10 [0.49 , 167.79]
GOG 0218	8	1215	5	601	22.1%	0.79 [0.26 , 2.41]
**Total (95% CI)**		**2716**		**2095**	**100.0%**	**2.45 [1.27 , 4.72]**
Total events:	67		23			

Heterogeneity: Tau² = 0.19; Chi² = 6.15, df = 4 (P = 0.19); I² = 35%
Test for overall effect: Z = 2.67 (P = 0.008)
Test for subgroup differences: Not applicable

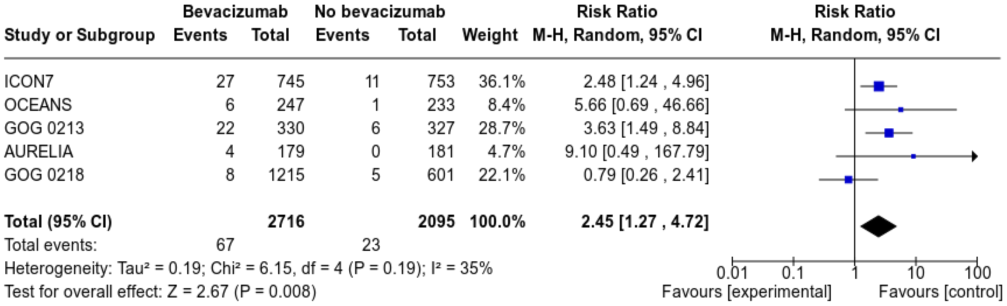

S2B: ATE risk in frontline setting

Study or Subgroup	Bevacizumab Events	Bevacizumab Total	No bevacizumab Events	No bevacizumab Total	Weight	Risk Ratio M-H, Random, 95% CI
ICON7	25	745	11	753	58.7%	2.30 [1.14 , 4.63]
GOG 0218	8	1215	5	607	41.3%	0.80 [0.26 , 2.43]
**Total (95% CI)**		**1960**		**1360**	**100.0%**	**1.49 [0.54 , 4.12]**
Total events:	33		16			

Heterogeneity: Tau² = 0.33; Chi² = 2.48, df = 1 (P = 0.12); I² = 60%
Test for overall effect: Z = 0.76 (P = 0.45)
Test for subgroup differences: Not applicable

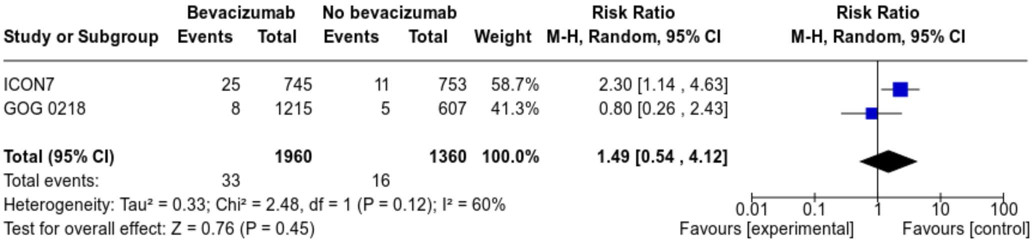

S2C: ATE risk in relapsed platinum-sensitive setting

Study or Subgroup	Bevacizumab Events	Bevacizumab Total	No bevacizumab Events	No bevacizumab Total	Weight	Risk Ratio M-H, Fixed, 95% CI
OCEANS	7	247	2	233	25.5%	3.30 [0.69 , 15.73]
GOG 0213	22	330	6	327	74.5%	3.63 [1.49 , 8.84]
Cong	0	0	0	0		Not estimable
NSGO-AVANOVA2/ENGOT-ov24	0	0	0	0		Not estimable
**Total (95% CI)**		**577**		**560**	**100.0%**	**3.55 [1.64 , 7.69]**
Total events:	29		8			

Heterogeneity: Chi² = 0.01, df = 1 (P = 0.92); I² = 0%
Test for overall effect: Z = 3.21 (P = 0.001)
Test for subgroup differences: Not applicable

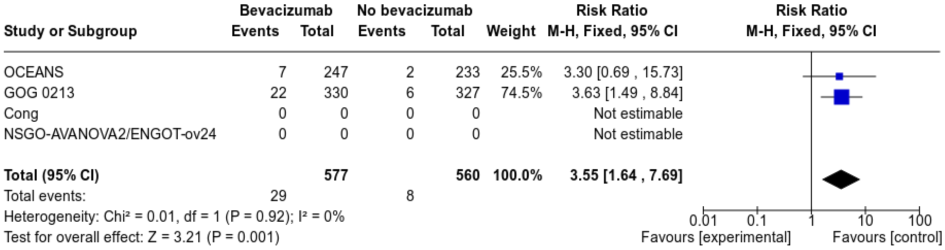

S2D: ATE risk in relapsed platinum-sensitive setting

Study or Subgroup	Bevacizumab Events	Bevacizumab Total	No bevacizumab Events	No bevacizumab Total	Weight	Risk Ratio M-H, Random, 95% CI
AURELIA	4	179	0	181	100.0%	9.10 [0.49 , 167.79]
Liu	0	0	0	0		Not estimable
**Total (95% CI)**		**179**		**181**	**100.0%**	**9.10 [0.49 , 167.79]**
Total events:	4		0			

Heterogeneity: Not applicable
Test for overall effect: Z = 1.49 (P = 0.14)
Test for subgroup differences: Not applicable

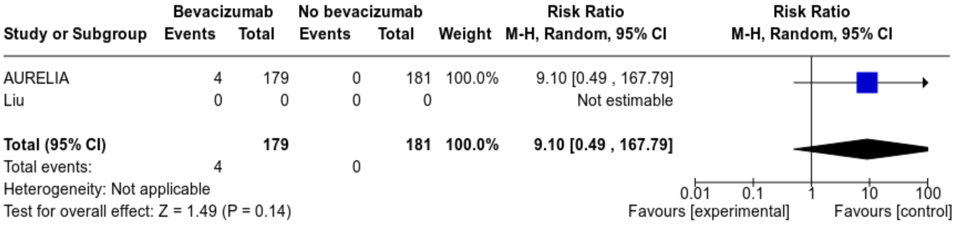

Figure S3: Risk of venous thromboembolic events (VTE) in various disease settings

S3A: VTE risk in all populations

Study or Subgroup	Bevacizumab Events	Bevacizumab Total	No bevacizumab Events	No bevacizumab Total	Weight	Risk Ratio M-H, Random, 95% CI
ICON7	50	745	31	753	35.9%	1.63 [1.05 , 2.52]
OCEANS	11	247	6	233	7.1%	1.73 [0.65 , 4.60]
GOG 0213	0	330	0	327		Not estimable
AURELIA	5	179	8	181	5.7%	0.63 [0.21 , 1.89]
GOG 0218	78	1215	35	601	45.8%	1.10 [0.75 , 1.62]
GEICO-1205	2	35	0	33	0.8%	4.72 [0.24 , 94.85]
ANTHALYA	6	58	2	37	2.9%	1.91 [0.41 , 8.98]
mEOC/GOG 0241	1	24	0	26	0.7%	3.24 [0.14 , 75.91]
NSGO-AVANOVA2/ENGOT-ov24	2	49	1	48	1.2%	1.96 [0.18 , 20.90]
**Total (95% CI)**		**2882**		**2239**	**100.0%**	**1.32 [1.02 , 1.72]**
Total events:	155		83			

Heterogeneity: Tau² = 0.00; Chi² = 5.09, df = 7 (P = 0.65); I² = 0%
Test for overall effect: Z = 2.10 (P = 0.04)
Test for subgroup differences: Not applicable

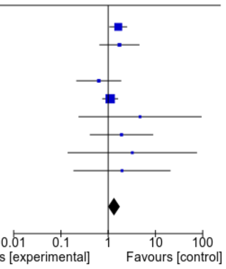

S3B: VTE risk in frontline setting

Study or Subgroup	Bevacizumab Events	Bevacizumab Total	No bevacizumab Events	No bevacizumab Total	Weight	Risk Ratio M-H, Random, 95% CI
ICON7	50	745	31	753	41.8%	1.63 [1.05 , 2.52]
GOG 0218	78	1215	35	601	53.2%	1.10 [0.75 , 1.62]
ANTHALYA	6	58	2	37	3.3%	1.91 [0.41 , 8.98]
mEOC/GOG 0241	1	24	0	26	0.8%	3.24 [0.14 , 75.91]
GEICO-1205	2	35	0	33	0.9%	4.72 [0.24 , 94.85]
**Total (95% CI)**		**2077**		**1450**	**100.0%**	**1.35 [1.02 , 1.79]**
Total events:	137		68			

Heterogeneity: Tau² = 0.00; Chi² = 2.94, df = 4 (P = 0.57); I² = 0%
Test for overall effect: Z = 2.09 (P = 0.04)
Test for subgroup differences: Not applicable

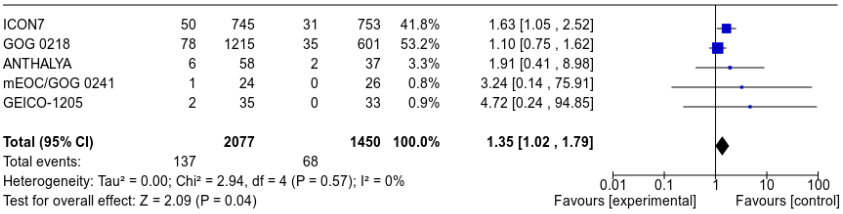

S3C: VTE risk in relapsed platinum-sensitive disease

Study or Subgroup	Bevacizumab Events	Bevacizumab Total	No bevacizumab Events	No bevacizumab Total	Weight	Risk Ratio M-H, Random, 95% CI
OCEANS	11	247	6	233	85.4%	1.73 [0.65 , 4.60]
GOG 0213	0	330	0	327		Not estimable
NSGO-AVANOVA2/ENGOT-ov24	2	49	1	48	14.6%	1.96 [0.18 , 20.90]
**Total (95% CI)**		**626**		**608**	**100.0%**	**1.76 [0.71 , 4.35]**
Total events:	13		7			

Heterogeneity: Tau² = 0.00; Chi² = 0.01, df = 1 (P = 0.92); I² = 0%
Test for overall effect: Z = 1.23 (P = 0.22)
Test for subgroup differences: Not applicable

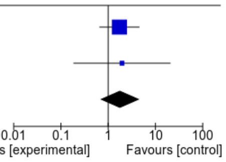

S3D: VTE risk in relapsed platinum-resistant disease

Study or Subgroup	Bevacizumab Events	Bevacizumab Total	No bevacizumab Events	No bevacizumab Total	Weight	Risk Ratio M-H, Random, 95% CI
AURELIA	4	179	8	181	100.0%	0.51 [0.15 , 1.65]
Liu	0	0	0	0		Not estimable
**Total (95% CI)**		**179**		**181**	**100.0%**	**0.51 [0.15 , 1.65]**
Total events:	4		8			

Heterogeneity: Not applicable
Test for overall effect: Z = 1.13 (P = 0.26)
Test for subgroup differences: Not applicable

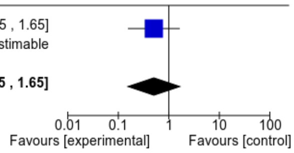

Supplement: Supplementary file 1 [file cancers-13-04603-s001.zip › cancers-1363373-Sl.pdf]
